# Ankle Fractures in Diabetic Patients: Report of Two Cases

**DOI:** 10.7759/cureus.13519

**Published:** 2021-02-23

**Authors:** Ioannis P Galanopoulos, Panagiotis Stavlas, Sofia M Voyaki, Spyridon A Psarakis

**Affiliations:** 1 Orthopedic Department, Thriasio General Hospital, Athens, GRC; 2 Orthopedic Department, Thriassio General Hospital, Athens, GRC; 3 Department of Internal Medicine, G. Gennimatas Athens General Hospital, Athens, GRC

**Keywords:** ankle fractures, diabetes mellitus

## Abstract

Although ankle fractures are very common cases for orthopedic surgeons with an easy diagnosis and very good outcomes either with conservative or with operative treatment, in diabetic patients, these fractures can be complex with difficult solutions. We report two cases with ankle fractures in diabetic patients from our department with demanding diagnostic or therapeutic approaches and poor outcomes. The first case, a 65-year-old man with undiagnosed diabetes mellitus and neglected ankle fracture dislocation because of diabetic neuropathy, underwent open reduction internal fixation with additional external fixation. Early after the operation, he presented with pin-tract infection, and later after the operation, he developed osteomyelitis which led to salvage below-knee amputation. In the second case, a 70-year-old woman with diabetes mellitus and severe coexisting medical comorbidities underwent open reduction internal fixation plus external fixation for an acute fracture-dislocation of the left ankle. Early after the operation, she developed ischemic lesions of the toes with worsened status despite the vascular surgeon's instructions. Although a below-knee amputation could be an acceptable choice, she denied it. As a result, systematic complications led to her death. It is very important for surgeons to follow an algorithm when they have to manage ankle injuries in diabetic patients because, in these patients, ankle fractures are very demanding and misdiagnosed cases with difficult treatment algorithms and often poor outcomes.

## Introduction

The fractures of the ankle joint are common injuries, most of the time easily diagnosed and treated either conservatively or surgically [1]. However, in diabetic patients, these fractures constitute demanding cases for both the diagnosis and the treatment [2]. We report two cases of bimalleolar fractures-dislocations of the ankle joint with difficult management and poor final outcome.

## Case presentation

Case 1

**Figure 1:**
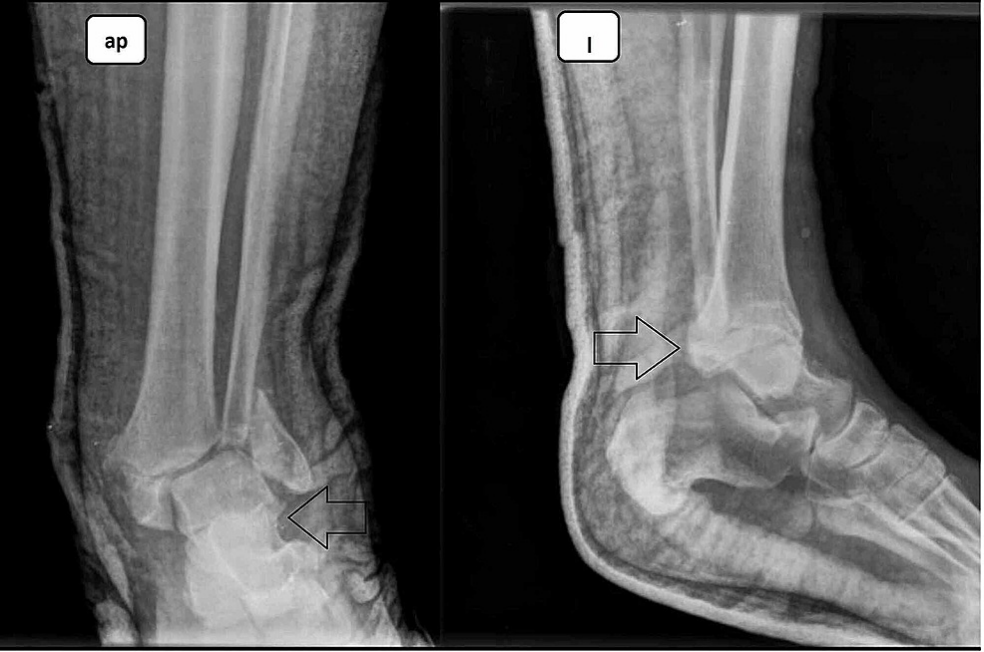
Neglected fracture with depression of the articular surface Anteroposterior (ap) and lateral (l) views.

**Figure 2:**
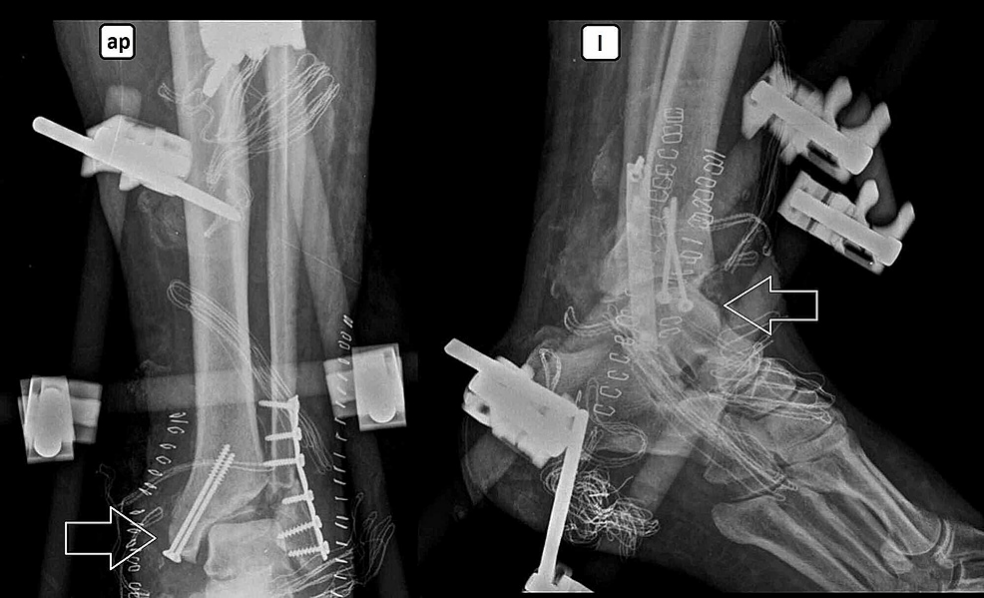
Open reduction internal fixation with additional external fixation: immediate postoperative image Anteroposterior (ap) and lateral (l) views.

A 65-year-old male presented to the emergency department of our hospital reporting an ankle joint injury twenty days before. Initially, he had swelling and no pain and walked normally. As the swelling did not get better, he decided to visit an orthopedic surgeon elsewhere. The surgeon diagnosed an ankle sprain and gave him instructions without radiographic investigations. Some days later, the patient noted a leg malalignment and discomfort during walking without skin lesions. After the X-ray imaging in our hospital, he was diagnosed with a bimalleolar fracture-dislocation with depression of the articular surface of the lower tibia (Figure 1). No medical comorbidities were reported. After the laboratory examinations, he was diagnosed with diabetes mellitus (type II). This could explain the mild symptoms and the painless walking after the injury. The plain radiographs were negative for Charcot arthropathy. The patient underwent open reduction internal fixation, but the joint remained unstable because of the articular depression of the distal tibia. An external fixation system was additionally used to stabilize the construct (Figure 2). The initial outcome was acceptable, and the patient remained hospitalized uneventfully for five days. Furthermore, a diabetes treatment protocol was introduced by endocrinologists. Three weeks after, he complained of pin loosening of one calcaneal half pin. We decided to remove the external fixation system in order to avoid deep infection. Per os, antibiotics were administered (second-generation cephalosporin), and the pin hole normally closed fifteen days after.

**Figure 3:**
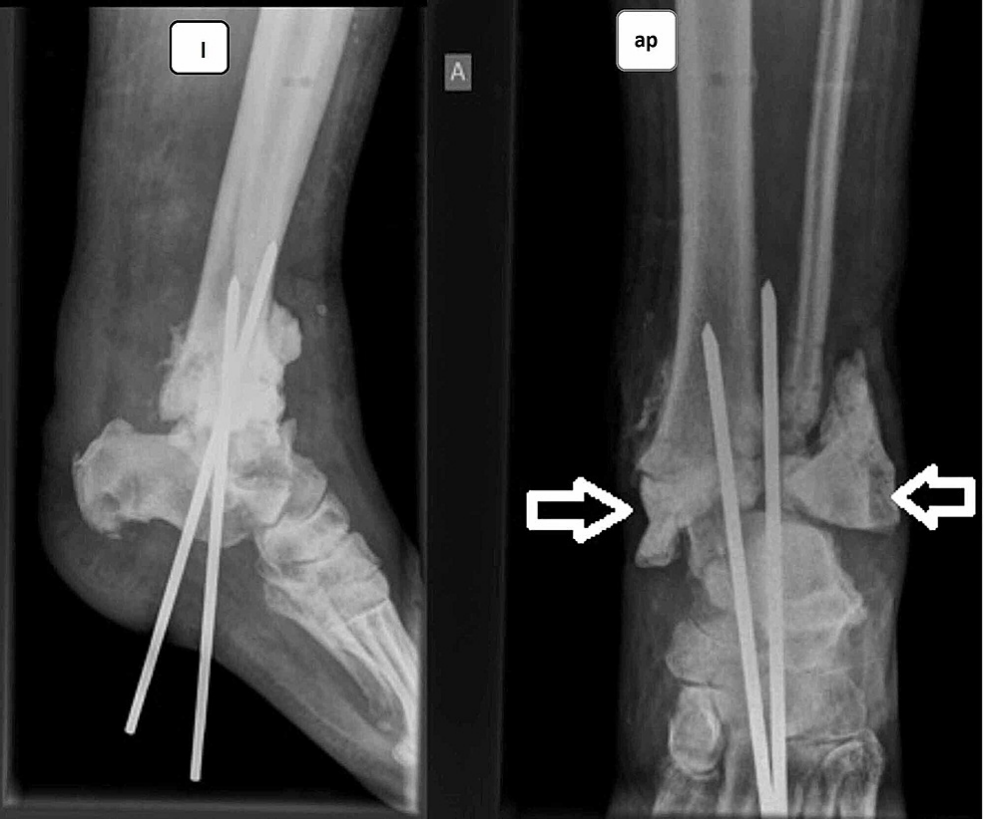
Radical debridement, bone cement filling the defects, temporary stabilization with Steinman pins Anteroposterior (ap) and lateral (l) views.

For the next two months, the patient lost his follow-up. He presented three months after the operation in septic shock (fever, tachycardia, visual illusions, increased urea and creatinine levels, C-reactive protein [CRP] - 280), with an open wound over the medial malleolus and pus drainage. He also had a large necrotic area over the dorsal surface of the foot because of a recent superficial injury. The ankle was absolutely unstable, and the lateral malleolar plate was broken. The patient underwent aggressive irrigation and debridement in the operating room. All the necrotic soft tissue and bone were removed, with the necrotic lesion of the dorsal foot included. The bone defect was filled with bone cement impregnated with vancomycin, and the joint was temporarily stabilized with two calcaneotalotibial Steinman pins (Figure 3). For the open wound of the dorsal foot, a vacuum drainage system was applied, and the patient started a course of intravenous antibiotics based on the antibiogram (S. aureus and Enterococcus spp). Although his vital signs returned to normal levels immediately after the surgery, one month postoperatively, the CRP level remained at a plateau level (110), and the toes presented ischemic lesions. After an extensive conversation with the patient, we proposed a below-knee amputation as the most reliable option in this phase. The patient underwent a below-knee amputation without any complication, and three weeks after, he had a good stump and began the preparation for the application of an artificial leg. Twelve months postoperatively, the patient has fully returned to his daily activities and his work. 

Case 2

**Figure 4:**
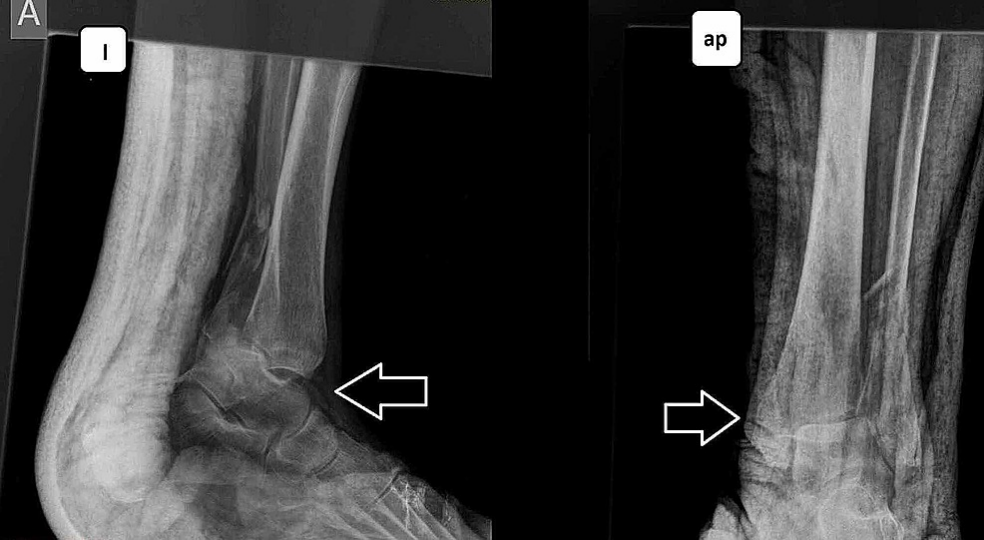
Ankle fracture-dislocation Anteroposterior (ap) and lateral (l) views.

A 74-year-old woman was admitted to the emergency department of our hospital after left ankle injury. The radiological investigation highlighted a comminuted fracture-dislocation of the left ankle with mild skin damage (Figure 4).

**Figure 5:**
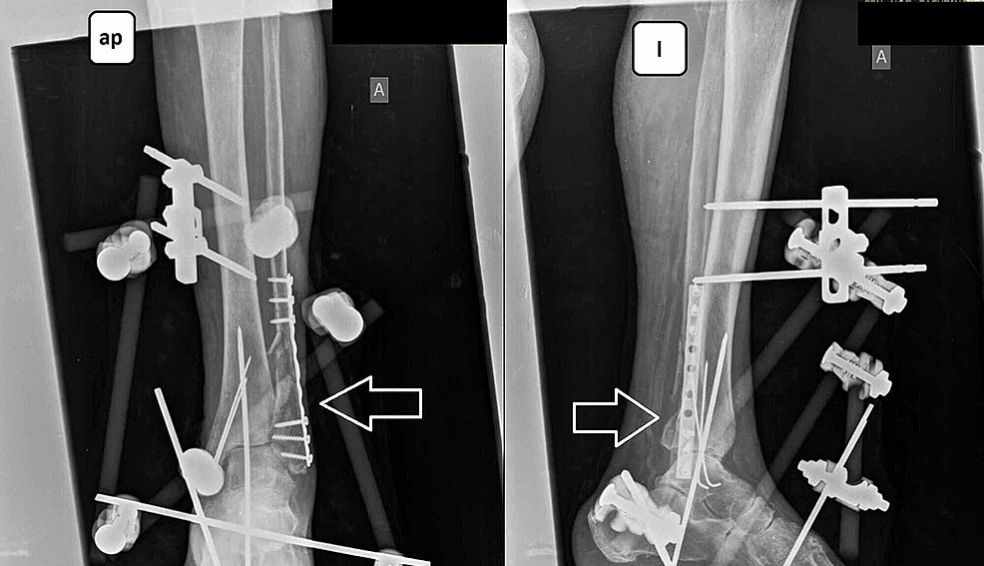
Open reduction internal fixation with additional external fixation: immediate postoperative outcome Anteroposterior (ap) and lateral (l) views.

Her medical history reported gastroesophageal reflux, atrial fibrillation, chronic renal disease under dialysis, and diabetes mellitus (type II). She underwent open reduction and internal fixation under regional anesthesia with additional external fixation for extra stabilization of the ankle joint (Figure 5).

Postoperatively she was treated with prophylactic low molecular weight heparin (LMWH) and antibiotics. Three days postoperatively, she presented with initial symptoms of vascular insufficiency of the first and second toes. Because of the ischemic lesions of the toes, vascular surgeons suggested a raised dose of LMWH up to therapeutic level and close follow-up. A renal specialist consultation was also asked for. Three days after, the patient decided to continue her treatment elsewhere. The orthopedic surgeons who undertook her treatment removed the external fixation device for better vascular function.

A month later, she had a new consultation from a vascular surgeon who suggested below-knee amputation because of the ongoing vascular insufficiency and the worsened soft tissue necrosis. The patient was also psychologically supported. Finally, the patient denied undergoing a salvage amputation, and some days after, she passed away because of systematic complications.

## Discussion

A higher rate of complications and cost of care makes ankle fractures in diabetic patients challenging cases for orthopedic surgeons. Given the fact that one-quarter of Americans over the age of 65 have a diagnosis of diabetes and 84.1 million over the age of 18 are pre-diabetic, ankle fractures in the diabetic population are not a rare entity [3]. The management of these cases gets even more demanding because diabetic patients tend to have other medical comorbidities such as end-stage renal disease (ESRD), peripheral vascular disease, coronary artery disease, neuropathy, and obesity. As a result, the mean hospital stay and healthcare cost of ankle fractures of diabetic patients are significantly higher than in non-diabetic patients. Significantly higher is also the rate of reoperation for irrigation and debridement and amputation in 100 and 135.5 days after the fracture averagely, according to Pincus et al. [4].

These patients have to be informed that they are more prone to postoperative complications when surgery is indicated. With open reduction internal fixation of closed malleolar fractures in insulin-dependent diabetic patients, the classic treatment has a 2.6 greater odds for early postoperative infection [5]. Alternative treatment options, such as trans-articular nailing, present fewer complications than traditional fixation methods in elderly patients with no significant decrease of functional level [6].

Concerning neglected cases, such as nonunion or malunion in nonoperatively treated patients, the complication rate is significantly higher than the cases with primary fixation, and the complication rate among nonoperatively treated patients raises up to 75% and, in some studies, up to 100%. Lovy et al. reported 100% of postoperative complications in case of second-time surgeries for neglected cases because diabetes often leads to impaired wound healing, delayed fracture healing, vasculopathy, neuropathy, and Charcot arthropathy [7]. In the literature, some authors suggest nonoperative treatment versus authors who follow operative treatment. This controversy depicts the disadvantages and the high complication rate for both options. The surgeons' most acceptable option seems to be the early operative fixation in these cases because both nonoperative and delayed operative treatment increase dramatically the complications [8, 9]. 

Even more demanding is the management of a failed operation of ankle fractures in diabetic patients. The limb salvage can be challenging, and no clear treatment algorithm exists. Complicated cases of ankle fractures in people with diabetes present longer hospitalization and greater hospital charges. Furthermore, many of these patients suffer also from peripheral neuropathy, and the risk of catastrophic complications is higher and can result even in limb loss. Additionally, diabetic peripheral neuropathy presents a unique problem in that seemingly innocuous injuries can lead to the initiation of Charcot neuroarthropathy [10].

The rigid internal fixation is the first-line treatment for unstable ankle fractures in people with diabetes. The internal fixation can be supported by additional methods like external fixation, multiple tetracortical screws, or the use of Kirschner wires or Steinmann pins across the tibiotalar joint for fixation strengthening and alignment maintaining [2].

Vaudreuil et al. reported that there were no patients who ended up with an amputation that did not present with infection after a failure of operative management. Furthermore, limb salvaging was feasible in patients with low preoperative infection rates (two of 14 patients). According to this study, the amputation group patients underwent followed repeated irrigation and debridement surgeries (six surgeries on average) because of deep infection before the amputation in contrast with the limb salvage group, in which the average irrigation and debridement surgeries was 3,5 [10]. 

In attempted limb salvage, rigid internal fixation is frequently indicated even in minimally displaced fractures to prevent the development of a Charcot event. Sometimes, another good option is the definitive fusion of the ankle joint [11].

It is very important for surgeons to follow an algorithm to manage ankle injuries in diabetic patients. Although complete guideline for the management of these cases does not exist,Yee et al. proposed a diagnostic and management algorithm that incorporates a quantitative scoring system in hope to achieve a practical approach to this complex and challenging problem (AFDA - Adelaide Fracture in the Diabetic Ankle Algorithm and Score) [12].

This algorithm differs from the algorithm for non-diabetic patients with ankle injuries. In our case, the patient visited an orthopedic surgeon immediately, but he did not suggest an X-ray imaging based on the history and the clinical picture. According to AFDA, the need for X-ray imaging in case of ankle injuries in people with diabetes is differentiated from the Ottawa ankle rules. Because of the possibility of an insensate lower limb, the patient’s history may be difficult to elicit, which occurred first: the fracture or acute Charcot’s. However, if any of these are present, the AFDA management algorithm and score can be applied.

The AFDA scoring system is based on assessable patient factors. Two points have been allocated to factors more readily seen in the notable publications to suggest either poor outcomes of standard internal fixation or better outcomes with rigid fixation/arthrodesis. A Semmes Weinstein monofilament 10 g/5.07 at the plantar aspect of either the great toe, first, third, or fifth metatarsal head can be used to assess the presence of neuropathy [13]. The presence of vasculopathy can be defined as peripheral oxygen saturations consistently less than 95% or an ankle-brachial index (ABI) of less than 0.65 [14]. Obesity can be defined as a BMI greater than 30. Smoking has not been included as a factor because it increases general surgical risk, independent of fixation technique.

## Conclusions

Ankle fractures in diabetic patients are very demanding and misdiagnosed cases with difficult treatment algorithms and often poor outcomes. The diagnostic approach of ankle joint injuries in diabetic patients needs to be detailed and radiographic investigation is necessary. The surgeon has to inform the patient of all the possible complications, especially when the fracture is neglected. Patients with secondary effects of diabetes mellitus also have a significant hazard of major complications even death. Salvage procedures like amputation, usually below the knee, can save lives, especially in the case of osteomyelitis or ischemic necrosis.
